# Drug-Coated Balloons for Treatment of Internal Carotid Artery Restenosis After Stenting: A Single-Center Mid-Term Outcome Study

**DOI:** 10.1007/s00270-024-03663-7

**Published:** 2024-02-07

**Authors:** Kamran Hajiyev, Hans Henkes, Ali Khanafer, Philipp Bücke, Florian Hennersdorf, Hansjörg Bäzner, Philipp von Gottberg

**Affiliations:** 1 Neuroradiologische Klinik, Klinikum Stuttgart, Stuttgart, Germany; 2 Medizinische Fakultät, Universität Duisburg-Essen, Essen, Germany; 3 Universitätsklinik für Neurologie, Bern University Hospital, Inselspital, Bern, Switzerland; 4 Abteilung Diagnostische und Interventionelle Neuroradiologie, Radiologische Universitätsklinik Tübingen, Tübingen, Germany; 5 Neurologische Klinik, Klinikum Stuttgart, Stuttgart, Germany

**Keywords:** Stroke, Carotid artery stenting, In-stent restenosis, Drug-coated balloons, Carotid artery atherosclerotic disease

## Abstract

**Purpose:**

Endovascular and surgical treatments of stenosis of the extracranial internal carotid artery (ICA) are common procedures, yet both introduce a risk of restenosis due to endothelial hyperplasia. Drug-coated balloons (DCBs) are designed to decrease neointimal hyperplasia, however rarely used in the neurovascular setting. This study retrospectively analyzes mid-term results of DCB-treated in-stent restenosis (ISR) of the ICA.

**Materials and Methods:**

The medical history, comorbidities, and periprocedural data of patients receiving DCB treatment for > 50% ISR of the ICA after carotid artery stenting were analyzed. Follow-up after DCB treatment was performed with Doppler ultrasound. Suspicious cases were checked with CT- or MR-angiography and—if there was agreement between the modalities—validated with digital subtraction angiography. Potential risk factors for restenosis and differences in outcomes after PTA with three types of DCB balloons were evaluated.

**Results:**

DCB treatment was performed in 109 cases, 0.9% of which involved in-hospital major stroke; no minor strokes occurred. A total of 17 patients (15.6%) had recurrent ISR after DCB treatment, after a mean time of 30.2 months (7–85 months). Tobacco use was significantly associated with a higher incidence of recurrent ISR.

**Conclusion:**

DCB angioplasty for ISR is an effective treatment that may delay and decrease restenosis. Treating comorbidities and adopting lifestyle changes may additionally help prevent ISR.

**Graphical Abstract:**

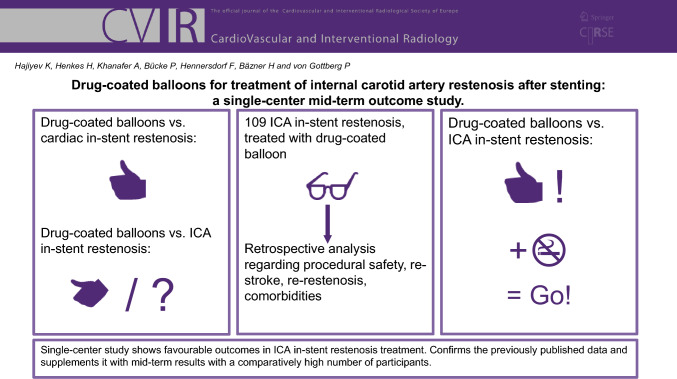

## Introduction

In the treatment of moderate- to high-grade stenoses of the ostium of the internal carotid artery (ICA), carotid artery stenting (CAS) is a common and established procedure with long-term results that are comparable to surgical options [1–3].

CAS may lead to proliferation to the vessel’s endothelium, and neointimal hyperplasia is believed to be a major factor influencing in-stent restenosis (ISR) [4]. In the CREST study, in which end points of death/stroke/myocardial infarction were analyzed in patients with ICA stenosis who were randomly assigned to receive CEA or CAS, patients with rather than without ICA ISR had a higher risk of recurrent stroke [5].

In a 2019 meta-analysis of more than 16,000 carotid interventions, the cumulative risk of > 70% restenosis has been found to be 5.2% at 12 months after CAS [6]. The 2018 International Carotid Stenting Study (ICSS) has reported a 40% five-year cumulative risk of restenosis after CAS. Again, restenosis significantly influenced the incidence of ipsilateral recurrent stroke in the study population [7].

Drug-coated balloons (DCBs) are devices specifically designed to challenge neointimal hyperplasia [8, 9].

In the neurovascular setting, after pioneering works by Vajda et al. in 2009 [10] and 2011 [11], and despite promising additional data in 2012 [12] and 2014 [13], further reports on DCB treatment of ISR have been sparse but remain promising [14–16].

To further elucidate the role of DCBs in the treatment of ISR, we analyzed mid-term results of ICA ISR treated with DCB.

## Methods

This single-center retrospective analysis was conducted in accordance with the Declaration of Helsinki and was approved by the local ethics committee. All patients were informed in detail about the specific use and off-label indications for DCBs in ISR, and written informed consent was obtained from all patients before the procedure.

### Patient Selection and Evaluation

Both symptomatic and asymptomatic patients with extracranial carotid artery stenosis (NASCET > 50%) confirmed by digital subtraction angiography and treated with CAS in an elective or acute setting between 2009 and 2023 were retrospectively analyzed.

Patients with suspected ISR > 50%, concordant on Doppler and CT-/MR-angiography imaging, after confirmation by digital subtraction angiography, were treated with conventional balloons or DCBs, with or without additional stent implantation. The appropriate treatment method (bare balloon vs. DCB vs. balloon angioplasty and re-stenting) was carefully considered by our neurovascular team, on the basis of the morphology and length of the ISR. Only patients with confirmed ISR > 50% and exclusive DCB treatment were included.

The pre-procedural evaluation included a neurological and degree –of –stenosis assessment and platelet function test (Multiplate®, Roche Diagnostics/VerifyNow®, Werfen). ISR was considered symptomatic if a patient experienced transient ischemic attacks (TIAs), amaurosis fugax or cerebral infarction of the corresponding ICA territory in the preceding six months, or acute cerebral ischemia in the preceding seven days.

Patients were considered asymptomatic if they had neither stroke nor TIA within the preceding six months. The NASCET method [17] was used to determine the degree –of –stenosis.

Baseline demographics; risk factors; and clinical and periprocedural data were retrospectively collected from our hospital’s database.

### Procedure and Technical Data

Patients received dual antiplatelet therapy for at least three days before the procedure, and general anesthesia (GA) was preferred. The aforementioned platelet function tests were performed on the day of the procedure to ensure adequate platelet inhibition. A 6 F guiding catheter was used for selective catheterization of the common carotid artery. Diagnostic angiography was performed to confirm the degree and morphology of the ISR. Unfractionated heparin was infused as a bolus of 3000–5000 IU. A 0.014-inch microguidewire was then navigated beyond the ISR. A DCB of the proper size, preferably a 4/20-mm balloon, was used to cover the whole length of the ISR. Oversizing was deliberately avoided. The DCB was kept inflated for 60–90 s, then deflated and withdrawn. A final angiogram was obtained in all cases. Technical success was defined as restoration of blood flow within the stent, with residual stenosis below 30%. The balloon catheters used were Emperor® (AR Baltic Medical, Vilnius, Lithuania; *n* = 7), SeQuent® Please (B. Braun SE, Melsungen, Germany; *n* = 50), and SeQuent® Please NEO (B. Braun SE, Melsungen, Germany; *n* = 52).

### Post-procedural Period

Arterial blood pressure should be maintained at a systolic level of 120–130 mmHg for at least 24 h. A neurological assessment and, in most cases, post-procedural computed tomography or magnetic resonance imaging (MRI) were performed before hospital discharge. Periprocedural neurological events were documented and categorized as follows:

- TIA: reversible focal neurological deficit < 3 h.
- Cerebral hyperperfusion syndrome: symptoms associated with brain edema and intracerebral/subarachnoid hemorrhage.
- Stroke: acute, persistent focal neurological deficit with cerebral ischemia; categorized as:
  - Major stroke – an increase on the mRS of ≥ 3 points.
  - Minor stroke – an increase on the mRS of ≤ 2 points from pre-stroke status.

All patients were scheduled for ISR checkup through Doppler sonographic imaging at 3, 6, 9, and 12 months after CAS, and then every six months thereafter. The occurrence of recurrent stenosis after DCB therapy was defined as the primary outcome.

### Statistical Analysis

Continuous data are described as the mean, median, minimum, and maximum. Hazard ratios with 95% confidence intervals (CIs) were estimated with Cox regression to analyze the influence of continuous data on survival time. Numbers and percentages were used to describe categorical data. The incidence rates of ISR were calculated as events per 100 years. Incidence rate ratios (IRRs) were calculated to compare the incidence rates between groups. For incidence rates and IRRs, 95% CIs are given. Equality of survivor functions was compared with log-rank tests. All statistical tests were two-sided and had a significance level of 0.05. Stata/IC 16.1 for Unix was used for the statistical analysis.

## Results

Among 3489 patients who underwent CAS in the mentioned period, 190 patients received treatment for ISR at our hospital. Exclusive treatment with DCB angioplasty was performed in 109 patients, all of which were technically successful and are reported herein.

This patient cohort included 38 women and 71 men, and the median age was 68 years (range: 32–86 years). The distribution of symptomatic vs. asymptomatic stenosis was *n* = 5 vs. *n* = 104 (95.4%).

The most common comorbidities were arterial hypertension (79.8%), dyslipidemia (49.5%), tobacco use (45%), and diabetes mellitus (40.4%; Table 1).

**Table 1:** Baseline demographics and risk factors

	Total (*n* = 109)
Sex	
Female	38 (34.9%)
Male	71 (65.1%)
Age (years)	
Median	68
Range	32–86
Atrial fibrillation	10 (9.2%)
Diabetes mellitus	44 (40.4%)
History of tobacco use	49 (45%)
Arterial hypertension	87 (79.8%)
Peripheral artery disease	24 (22%)
Coronary artery disease	29 (26.6%)
Dyslipidemia	54 (49.5%)

Hundred-seven procedures (98.2%) were performed under GA. Thirty-five patients (32.1%) had an ISR exceeding 75%, and eight patients (7.3%) had a history of radiotherapy to the neck (Table 2).

**Table 2:** ICA-stenosis details at presentation for initial angioplasty

	Total (*n* = 109)
Location of ISR	
Right	51 (46.8%)
Left	58 (43.2%)
NASCET (%)	
50–75%	74 (67.9%)
> 75%	35 (32.1%)
Previous neck radiation	8 (7.3%)

One patient (0.9%) suffered a major stroke due to intraprocedural embolic M1 occlusion after ipsilateral DCB angioplasty, although the thrombus was immediately removed by mechanical thrombectomy.

No other neurological event and no myocardial infarction was observed in the in-hospital phase. A total of 68 patients underwent MRI after treatment, which revealed clinically inapparent microlesions on diffusion-weighted imaging (DWI) in 23 patients (33.8%). Three (2.8%) patients had minor complications at the femoral access site.

**Fig. 1:**
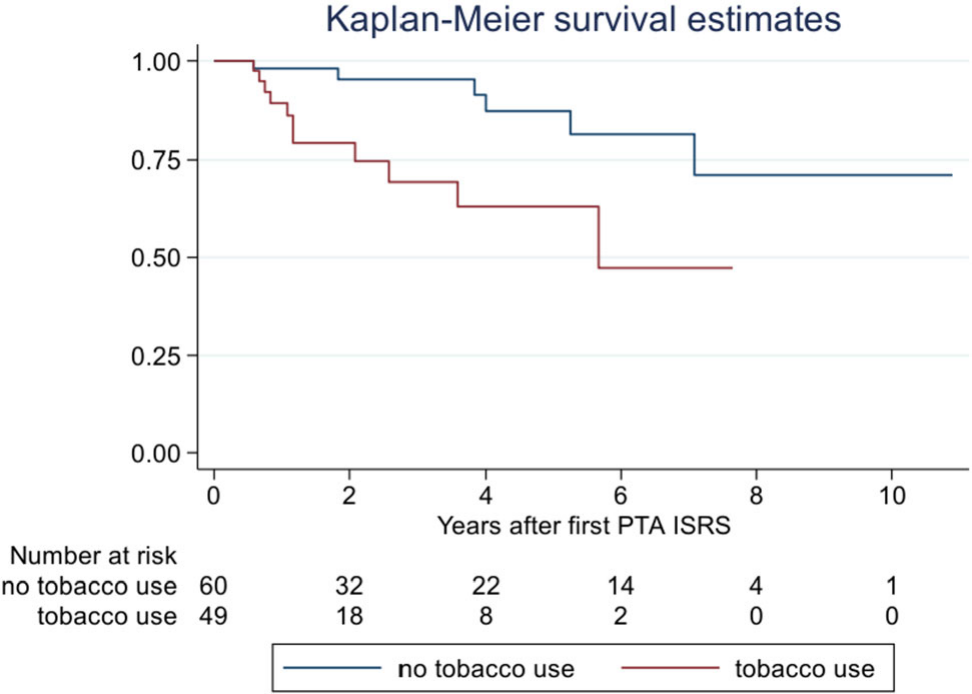
Estimated rates of patients without recurrent ISRS in relation to tobacco use

The primary outcome of recurrent ISR exceeding 50% occurred in 17 patients (15.6%) after a mean time of 30.2 months (7–85 months). All of these patients required follow-up treatment due to symptomatic ISRS (*n* = 12) or recurrent ISR > 75% (*n* = 5). Tobacco use (11/17, 64.7%) showed a statistically significant association with recurrent ISR (*p* = 0.005; Fig. 1). Five of the eight included patients with a history of neck radiation were represented in that group. Regarding the different balloon models, the mean time after re-intervention for the recurrent ISR was longer in patients treated with SeQuent® Please NEO, but the difference was not statistically significant. Furthermore, there were no significant differences in the results between the individual balloon models (Tables 3, 4).

**Table 3:** Characteristics and procedural data for patients with recurrent ISR

	Total (*n* = 17)
Sex	
Female	4 (23.5%)
Male	13 (76.5%)
Age (years)	
Median	68
Range	53–78
Atrial fibrillation	1 (5.9%)
Diabetes mellitus	10 (58.8%)
History of tobacco use	11 (64.7%)
Arterial hypertension	14 (82.4%)
Peripheral artery disease	1 (5.9%)
Coronary artery disease	5 (29.4%)
Dyslipidemia	8 (47.1%)
Time from DCB treatment to recurrent ISR (months)	
Mean	30.2
Range	7–85
Emperor®	
*n*	2
Mean	36.5
Range	25–48
SeQuent® Please	
*n*	9
Mean	24.1
Range	7–63
SeQuent® Please NEO	
*n*	6
Mean	37.2
Range	8–85

**Table 4:** Analysis of the influence of risk profiles comprising specific comorbidities on the prevalence of recurrent ISR

	*n*	ATO* (y)	ISR (*n*)	Incidence per 100 years (95% CI)	*p* value** IRR (95% CI)
Sex					
Female	38	102.6	4	3.90 (1.46; 10.38)	0.436
Male	71	198.8	13	6.54 (3.80; 11.26)	1.68 (0.52; 7.06)
Atrial fibrillation					
No	99	274.6	16	5.83 (3.57; 9.51)	0.699
Yes	10	26.9	1	3.72 (0.52; 26.42)	0.64 (0.02; 4.11)
Diabetes mellitus					
No	65	152.3	7	4.60 (2.19; 9.64)	0.484
Yes	44	149.2	10	6.70 (3.61; 12.46)	1.46 (0.50; 4.51)
Tobacco use					
No	60	203.0	6	2.96 (1.33; 6.58)	0.005
Yes	49	98.5	11	11.17 (6.18; 20.16)	3.78 (1.28; 12.44)
Arterial hypertension					
No	22	56.6	3	5.30 (1.71; 16.44)	0.910
Yes	87	244.9	14	5.72 (3.39; 9.65)	1.08 (0.30; 5.85)
Peripheral artery disease					
No	85	239.8	16	6.67 (4.09; 10.89)	0.126
Yes	24	61.7	1	1.62 (0.23; 11.50)	0.24 (0.01; 1.56)
Coronary artery disease					
No	80	205.8	12	5.83 (3.31; 10.27)	0.773
Yes	29	95.7	5	5.23 (2.18; 12.56)	0.90 (0.25; 2.73)
Dyslipidemia					
No	55	145.2	9	6.20 (3.23; 11.92)	0.753
Yes	54	156.3	8	5.12 (2.56; 10.23)	0.83 (0.28; 2.41)
Balloon					
Emperor®	7	20.6	2	9.69 (2.42; 38.74)	–
SeQuent® Please	50	193.6	9	4.65 (2.42; 8.94)	0.509***
SeQuent® Please NEO	52	87.3	6	6.87 (3.09; 15.30)	1.48 (0.43; 4.65)**

*Accumulated time of observation

**Log-rank test for equality of survivor functions

***Comparison of SeQuent® Please vs. SeQuent® Please NEO

*IRR* incidence rate ratio

## Discussion

The aim of this study was to investigate the effect of DCB on ISR in patients after CAS with regard to time –to –restenosis after DCB angioplasty and safety/periprocedural aspects. Published data on ICA ISR treated with DCBs are sparse, and studies covering higher patient numbers have shorter mean follow-up times compared to the mean follow-up time of 30.2 months in this study [18–20]. However, with 15.6% recurrence of ISR > 50% at a mean time –to –restenosis after DCB angioplasty of 32.6 months, results from this study are comparable with data found in the literature (0–23% restenosis > 50%; mean time –to –restenosis 23 months).

The differences between the mean time –to –restenosis of the studies and our data can possibly be explained by the different numbers of cases and follow-up periods as well as the selection of patients.

Regarding patient selection, a report published by Wu et al. [16] shows promising results for DCB treatment of patients with post-radiation stenosis of the ICA. Radiation-induced atherosclerosis of the ICA is a fast-progressing, aggressive form of vessel disease compared with lifestyle-induced atherosclerosis [21]. We observed a remarkably high proportion of patients with neck irradiation with re-restenosis after DCB treatment of the ICA (62.5%). Yet, the mean time –to –re-restenosis was nearly twice the entire follow-up time reported by Wu et al.: 12 months of follow-up vs. 22.8 months until re-restenosis after DCB treatment in our data. The pathomechanism underlying this type of ICA stenosis might be more sensitive to drug-eluting devices than plain devices, because of the high proportion of neointimal hyperplasia; consequently, treating radiation-induced ICA restenosis with DCBs may be beneficial.

The statistical significance of the association between continuous tobacco use and the development of recurrent ISR suggests that past and/or current tobacco use and a history of radiotherapy to the neck increase the risk of ISR, but may also indicate that addressing neointimal hyperplasia through DCB treatment may be only part of the solution. Lifestyle modification and management of comorbidities may therefore influence the risk of ISR after DCB treatment [22]. However, particularly in smokers with a history of radiotherapy to the neck, short-term checks for ISR may be advisable.

All applied DCBs had a paclitaxel coating; however, the doses and excipients differed: 2.2 μg/mm^2^ and dextran as excipient for the Emperor® DCB (as stated by the manufacturer) and 3 μg/mm^2^ for the SeQuent® Please [23] and SeQuent® Please NEO DCB with iopromide as excipient (as stated by the manufacturer). Excipients enhance the amount of drug transferred to the vessel wall. However, the extent of drug transfer varies considerably among different excipients/models of DCBs [24]. Thus, the differing results for the applied devices might be explained by different degrees of drug transfer or different doses in the device coating.

Regarding periprocedural safety, the 0.9% incidence of major stroke and no death suggest that DCBs can be considered safe. The majority of procedures were performed in GA. In CAS, local anesthesia is believed to be responsible for the lower rate of myocardial infarction in comparison with patients receiving open surgery in GA [25]. However, for DCB treatment, we prefer GA to achieve better patient tolerance to longer balloon inflation times and higher precision in stenosis treatment through machine-assisted breath-holding. It is also easier in GA to handle sudden fluctuations in blood pressure and epileptic seizures caused by the inflation of the balloon.

Coating wash-off in the vascular system distal to the treated lesion has been reported [26]. Regarding paclitaxel doses, a systemic dose of approximately 300 mg is reported to cause peripheral neuropathy in oncology. This dose greatly exceeds the total amount of paclitaxel in the coating of a DCB; to date, there are no reports on pharmacological effects of paclitaxel after DCB treatment in the neurovascular setting [27]. Consistent with this, no patients in this study reported symptoms or effects that could be attributed to the use of DCBs in a vessel directly supplying the brain. A definitive answer to the question of whether and to what extent the brain is harmed by the use of a DCB in a vessel directly supplying it can therefore not yet be given.

The limitations of this study are the limited comparability with previously published studies on the treatment of ICA ISR by DCB and the paucity of data published on this topic to date. In addition, this study lacked a control group, thus decreasing the comparability of the results. Another limiting factor of the study is the fact that a small proportion of patients did not undergo imaging after the procedure; however, this was due to uneventful procedures in which patients had no new symptoms and therefore probably only had minor impact on the results.

## Conclusion

DCB is beneficial in the treatment of ICA ISR in terms of the time-to-restenosis and may therefore decrease the risk of stroke recurrence; the effect may vary between the different DCB models due to the different dosage of the drug and the excipients.

However, DCB treatment may be only part of strategies to prevent restenosis, and lifestyle changes, particularly tobacco cessation, may also play a role.

## References

[1] Brott TG, Calvet D, Howard G (2019). Long-term outcomes of stenting and endarterectomy for symptomatic carotid stenosis: a preplanned pooled analysis of individual patient data. Lancet Neurol.

[2] Halliday A, Bulbulia R, Bonati LH (2021). Second asymptomatic carotid surgery trial (ACST-2): a randomised comparison of carotid artery stenting versus carotid endarterectomy. Lancet.

[3] Rosenfield K, Matsumura JS, Chaturvedi S (2016). Randomized trial of stent versus surgery for asymptomatic carotid stenosis. N Engl J Med.

[4] Papafaklis MI, Chatzizisis YS, Naka KK, Giannoglou GD, Michalis LK (2012). Drug-eluting stent restenosis: effect of drug type, release kinetics, hemodynamics and coating strategy. Pharmacol Ther.

[5] Lal BK, Beach KW, Roubin GS (2012). Restenosis after carotid artery stenting and endarterectomy: a secondary analysis of CREST, a randomised controlled trial. Lancet Neurol.

[6] Clavel P, Hebert S, Saleme S, Mounayer C, Rouchaud A, Marin B (2019). Cumulative incidence of restenosis in the endovascular treatment of extracranial carotid artery stenosis: a meta-analysis. J NeuroInterventional Surg.

[7] Bonati LH, Gregson J, Dobson J (2018). Restenosis and risk of stroke after stenting or endarterectomy for symptomatic carotid stenosis in the International Carotid Stenting Study (ICSS): secondary analysis of a randomised trial. Lancet Neurol.

[8] Axel DI, Kunert W, Göggelmann C (1997). Paclitaxel inhibits arterial smooth muscle cell proliferation and migration in vitro and in vivo using local drug delivery. Circulation.

[9] Piscione F, Piccolo R, Cassese S, Galasso G, Chiariello M. Clinical impact of sirolimus-eluting stent in ST-segment elevation myocardial infarction: a meta-analysis of randomized clinical trials. Catheter Cardiovasc Interv. Published online 2009:NA–NA. doi:10.1002/ccd.22017 10.1002/ccd.22017 19360858

[10] Vajda Z, Miloslavski E, Güthe T (2009). Treatment of stenoses of vertebral artery origin using short drug-eluting coronary stents: improved follow-up results. Am J Neuroradiol.

[11] Vajda Z, Güthe T, Aguilar Perez M (2011). Neurovascular in-stent stenoses: treatment with conventional and drug-eluting balloons. Am J Neuroradiol.

[12] Liistro F (2012). Drug-eluting balloon angioplasty for carotid in-stent restenosis’. J Endovasc Ther..

[13] Gandini R (2014). Long-term results of drug-eluting balloon angioplasty for treatment of refractory recurrent carotid in-stent restenosis. J Endovasc Ther..

[14] Pohlmann C, Höltje J, Zeile M, Bonk F, Urban PP, Brüning R (2018). Recurrent stenosis following carotid artery stenting treated with a drug-eluting balloon: a single-center retrospective analysis. Neuroradiology.

[15] Bhatia K, Akhtar IN, Akinci Y (2020). Drug-eluting balloon angioplasty for in-stent restenosis following carotid artery stent placement. J Neuroimaging.

[16] Wu CH, Lin TM, Chung CP, et al. Prevention of in-stent restenosis with drug-eluting balloons in patients with postirradiated carotid stenosis accepting percutaneous angioplasty and stenting. J NeuroInterventional Surg. Published online March 13, 2023:jnis-2022-019957. 10.1136/jnis-2022-019957 10.1136/jnis-2022-019957 PMC10804009 36914246

[17] Beneficial Effect of Carotid Endarterectomy in Symptomatic Patients with High-Grade Carotid Stenosis. *N Engl J Med*. 1991;325(7):445–453. 10.1056/NEJM199108153250701 10.1056/NEJM199108153250701 1852179

[18] Piccoli G, Biondi-Zoccai G, Gavrilovic V (2015). Drug-coated balloon dilation before carotid artery stenting of post-carotid endarterectomy restenosis. J Endovasc Ther.

[19] Tekieli Ł, Musiałek P, Kabłak-Ziembicka A, et al. Severe, recurrent in-stent carotid restenosis: endovascular approach, risk factors. Results from a prospective academic registry of 2637 consecutive carotid artery stenting procedures (TARGET-CAS). Adv Interv Cardiol. 2019;15(4):465–71. 10.5114/aic.2019.90221 10.5114/aic.2019.90221 PMC6956450 31933663

[20] Haupert G, Ammi M, Hersant J (2020). Treatment of carotid restenoses after endarterectomy: a retrospective monocentric study. Ann Vasc Surg.

[21] Xu J, Cao Y (2013). Radiation-induced carotid artery stenosis: a comprehensive review of the literature. Interv Neurol.

[22] Mihály Z, Vértes M, Entz L, Dósa E (2021). Treatment and predictors of recurrent internal carotid artery in-stent restenosis. Vasc Endovascular Surg.

[23] Ho HH, Ooi YW, Loh KK (2013). Clinical efficacy and safety of sequent please paclitaxel-eluting balloon in a real-world single-center registry of south-east asian patients. IJC Heart Vessels.

[24] Cortese B, Granada JF, Scheller B (2016). Drug-coated balloon treatment for lower extremity vascular disease intervention: an international positioning document. Eur Heart J.

[25] Dakour-Aridi H, Rizwan M, Nejim B, Locham S, Malas MB (2019). Association between the choice of anesthesia and in-hospital outcomes after carotid artery stenting. J Vasc Surg.

[26] Speck U, Stolzenburg N, Peters D, Scheller B (2016). How does a drug-coated balloon work? Overview of coating techniques and their impact. J Cardiovasc Surg (Torino).

[27] Margolis J, McDonald J, Heuser R (2007). Systemic nanoparticle paclitaxel (nab-Paclitaxel) for in-stent restenosis I (SNAPIST-I): a first-in-human safety and dose-finding study. Clin Cardiol.

